# Association of exclusive breast feeding and early cereal introduction with the development of Type-1 diabetes mellitus (T1DM)

**DOI:** 10.12669/pjms.42.3.13063

**Published:** 2026-03

**Authors:** Muhammad Saqib Manzoor, Muhammad Zulfiqar Siddiq, Perwez Ali, Asim Khurshid

**Affiliations:** 1 Muhammad Saqib Manzoor, Department of Paediatric Medicine, The Children’s Hospital, Institute of Child Health Multan, Pakistan; 2 Muhammad Zulfiqar Siddiq, Department of Paediatric Medicine, The Children’s Hospital, Institute of Child Health Multan, Pakistan; 3 Perwez Ali Department of Paediatric Medicine, Bakhtawar Amin Medical and Dental College Multan, Multan - Pakistan; 4 Asim Khurshid, Department of Paediatric Medicine, The Children’s Hospital, Institute of Child Health Multan, Pakistan

**Keywords:** Type-I diabetes mellitus, Breast feeding, Mumps

## Abstract

**Objective:**

To determine the association of exclusive breast feeding and early cereal introduction with the development of Type-I diabetes mellitus in children.

**Methodology:**

This case-control study was conducted at Pediatric Medicine Department of CH & ICH Multan, from March 2024 to August 2024. A total of 486 children aged 2-12 years (243 with T1DM and 243 controls) were consecutively enrolled. Data on the mode of delivery, duration of breastfeeding, timing of cereals introduction, history of mumps and family history of diabetes was collected. Logistic regression analysis was run to determine the association of exclusive breast feeding and early cereal introduction with the development of T1DM. Crude and adjusted odds ratio with 95% confidence interval are reported and p-value < 0.05 was taken significant.

**Results:**

The median age was seven years (IQR 5-9), with 58.8% females, 50.4% from rural areas, and 51.2% delivered by C-section. Median breastfeeding was for four months (IQR 2-5) and 20.6% were exclusively breastfed. History of mumps, early cereal introduction and family history of diabetes were reported in 8.6%, 47.1% and 17.5% respectively. Compared to controls, cases had higher odds of non-exclusive breastfeeding (OR 2.1; 95% CI 1.3-3.2), early cereal introduction (OR 2.7; 95% CI 1.9-3.9), female gender (OR 1.9; 95% CI 1.3-2.7), mumps history (OR 6.8; 95% CI 2.8-16.6) and positive family history of diabetes (OR 8.3; 95% CI 4.4-15.7).

**Conclusion:**

Lack of exclusive breastfeeding, early cereal introduction, female gender, history of mumps and family history of diabetes are independent risk factors for T1DM in children suggesting targeted preventive strategies in at risk populations.

## INTRODUCTION

Type-I diabetes mellitus (T1DM) is featured by hyperglycemia and an insulin shortage. It usually first appears in childhood and is produced by autoimmune or non-autoimmune factors that destroy the beta cells in the pancreas.^1^ In people who are genetically susceptible (human leukocyte antigen or HLA groups at risk), environmental variables like viruses, toxins, emotional stress, and others can exacerbate autoimmunity.^2^ Clinical signs of diabetes appear when reserves of beta-cells reduce by 90%.^3^ Food antigens are one of many environmental elements that have been reported to contribute to the condition’s development.^4^

Numerous studies on the impact of nutrition on Type-I diabetes during pregnancy and the first few years of life have produced conflicting results. Probiotic supplements, zinc supplements, vitamin D, vitamin C, and breastfeeding have all been shown to be protective against Type-I diabetes.^5^,^6^ Early consumption of eggs, vegetables, and gluten, however, may increase the risk.^7^

In addition to the psychological impact, children with diabetes also face socioeconomic repercussions that impact their families and entire communities. Their quality of life is further negatively impacted by frequent co-morbidities. The aim of this study was to ascertain how early cereal introduction and exclusive breastfeeding (EBF) affect the development of T1DM in children in our community. Authorities will be better able to plan and execute preventive measures to lessen the disease’s burden if they are aware of the modifiable risk factors from environment responsible for development of for Type-I diabetes.

## METHODOLOGY

This case-control study was performed at Pediatric Medicine Department of CH & ICH Multan over a period of 6-months from March 2024 to August 2024.A total of 243 children, 2 - 12 years of age, either male or female gender, diagnosed with Type-I diabetes mellitus (cases), from in-patient department and pediatric endocrinology outpatient department were consecutively enrolled in the study. Children without T1DM (controls) were selected from outpatient department. Informed consent was provided by the parents. Children were excluded if diagnosed with T1DM within first year of life (monogenic).

### Ethical Approval:

It was obtained from the ethics review committee approval (ERC# 124/CH&ICH Multan; Dated: 18.01.2024).

Data on age, gender, area of residence, mode of delivery, duration of exclusive breast feeding, early cereal introduction, history of mumps and family history of diabetes mellitus was collected by the research assistants not aware of T1DM status of the children. A sample size of 486 children (243 cases and controls each) was calculated using WHO sample size calculator taking prevalence of exclusive breastfeeding as 13.9% in cases and 22.6% in controls at 80% power of the study and 5% significance level.^8^ The data analysis was done through SPSS version 25. Descriptive statistics are presented in the form of median (IQR) and frequency/ percentages for quantitative and qualitative data respectively. Association of exclusive breast feeding and early cereal introduction with the development of T1DM is assessed through logistic regression analysis. Factors with a p-value of < 0.20 at bivariate analysis were entered in stepwise forward multivariable model. Crude and adjusted odds ratios with 95% confidence interval are reported and p-value of < 0.05 was considered significant for independent predictors of T1DM.

## RESULTS

The median (IQR) age of the subjects was seven (5 - 9) years and there were 58.8% (n=286) female children. Almost half (50.4%) belonged from rural areas and 51.2% (n=249) were delivered through cesarean section. Median duration of EBF was four (2 - 5) months and only 20.6% were exclusively breastfed. History of mumps, early cereal introduction and family history of diabetes were present in 8.6% (n=42), 47.1% (n=229) and 17.5% (n=85) participants. Age, area of residence, mode of delivery and duration of breast feeding were comparable between the case and controls. Frequency of female children (56.6% vs. 43.4%), history of mumps (85.7% vs. 14.3%), family history of diabetes (85.9% vs. 14.1%) and early cereal introduction (62.9% vs. 37.1%) were markedly higher in cases in contrast to controls. However, exclusive breastfeeding was substantially lower in cases in comparison to controls (36% vs. 64%) (Table-I).

**Table-I:** Characteristics of children with Type-I diabetes and healthy controls (N=486).

Characteristics	Overall (N=486)	Cases (n=243)	Control (n=243)	p-value*
Age (years)	7 (5 - 9)	7 (5 - 9)	7 (6 - 9)	0.592
Gender - Female	286 (58.8)	162 (56.6)	124 (43.4)	< 0.001
Residence - Rural	245 (50.4)	123 (50.2)	122 (49.8)	0.928
Delivery - cesarean	249 (51.2)	125 (50.2)	124 (49.8)	0.928
Breast feeding duration (months)	4 (2 - 5)	4 (3 - 5)	4 (2 - 7)	0.914
Exclusive breast fed - Yes	100 (20.6)	36 (36)	64 (64)	0.002
History of Mumps - Yes	42 (8.6)	36 (85.7)	6 (14.3)	< 0.001
History of Diabetes - Yes	85 (17.5)	73 (85.9)	12 (14.1)	< 0.001
Early cereal introduction - Yes	229 (47.1)	144 (62.9)	85 (37.1)	< 0.001

* Mann-Whitney U test for median comparison, chi-square test for categorical comparison.

Compared to controls the cases had 2.1 (1.3 - 3.2) odds of not exclusively breastfed, 2.7 (1.9 - 3.9) odds of early cereal introduction, 1.9 (1.3 - 2.7) odds of being female child, 6.8 (2.8 - 16.6) odds of positive mumps history and 8.3 (4.4 - 15.7) odds of positive family history of diabetes mellitus. Five independent predictors of Type-I diabetes mellitus were no exclusive breast feeding, early cereal introduction, female gender, positive history of mumps and family history of diabetes mellitus. Hosmer-Lemeshow indicated good model fitness (p-value = 0.183), and the model sensitivity, specificity, positive predictive value (PPV), negative predictive value (NPV) and accuracy were 60.1%, 80.7%, 75.7%, 66.9% and 70.4% respectively (Table-II).

**Table-II:** Association of exclusive breast feeding and early cereal introduction with the development of Type-I diabetes mellitus (N=486).

Characteristics	OR (95% CI)	p-value	aOR (95% CI)	p-value
Exclusive breast feeding - No	2.1 (1.3 - 3.2)	0.002	2.1 (1.3 - 3.6)	0.004
Early cereal introduction - Yes	2.7 (1.9 - 3.9)	< 0.001	2.6 (1.7 - 3.9)	<0.001
Age: > 5-years	0.7 (0.5 - 1.1)	0.122	--	--
Gender: Female	1.9 (1.3 - 2.7)	< 0.001	1.8 (1.2 - 2.7)	0.006
Area of residence: Urban	0.9 (0.7 - 1.4)	0.928	--	--
Mode of delivery: cesarean	1.0 (0.7 - 1.5)	0.928	--	--
Mumps: Yes	6.8 (2.8 - 16.6)	< 0.001	6.2 (2.4 - 15.8)	<0.001
Family h/o diabetes: Yes	8.3 (4.4 - 15.7)	< 0.001	8.1 (4.1 - 15.8)	<0.001

OR - Odds ratio for univariate model, aOR - adjusted odds ratio for multivariable model Hosmer-Lemeshow Goodness-of-fit test chi-square: 8.84, p-value = 0.183 Sensitivity: 60.1%, Specificity: 80.7%, PPV: 75.7%, NPV: 66.9%, Accuracy: 70.4%.

## DISCUSSION

One crucial step in preventing T1DM is the removal of avoidable environmental risk factors. Nevertheless, it is unclear exactly which characteristics are important and when and under what circumstances they should be removed.^9^,^10^ In this study, we looked at potential avoidable environmental factors and triggers, particularly early and exclusive breastfeeding and early introduction of cereal diet. We found that exclusive breastfeeding was significantly lower in cases in comparison to controls.

According to Cicekli I et al, the risk of T1DM decreased with each month when the duration of EBF was increased, but not with total breastfeeding.^8^ Another pooled analysis showed that Overall, after EBF for more than two weeks (20 studies; OR = 0.75, 95% CI 0.64-0.88), the incidence of diabetes decreased; however, the link was less pronounced after EBF for more than three months (30 studies; OR = 0.87, 95% CI 0.75-1.00).^11^ According to Çarkçı and Altuğ’s research, the proportion of children with T1DM who were exclusively breastfed for the first six months was 62.1%.^12^ It has been proposed that breastfeeding protects newborns by lowering intestinal permeability.^9^

Our finding that there were disproportionately more female children with T1DM is in line with current research studies on gender-related patterns. According to a Swedish registry study, there is a modest female predominance in T1DM with an earlier onset, especially after age five, which may be due to immunological or hormonal factors.^13^ In line with our observed gender trend towards female cases, the TEDDY investigation also found that girls had somewhat greater autoantibody development rates,^14^ indicating that female immune responses may speed up β-cell autoimmunity. The much greater incidence of mumps in T1DM patients is consistent with emerging data that connects viral triggers to beta-cell autoimmunity. Children with a history of mumps infection acquired Type-I diabetes almost a third of a decade earlier than their peers who were not infected, according to a Brazilian cohort.^15^ Research has also demonstrated that the mumps virus can infect pancreatic islets and alter HLA expression, which favors the idea that children who are genetically prone may develop T1DM as a result of viral infection.^16^ Therefore, our results suggest that mumps may be a contributing factor.

The excessive family history of diabetes in our cases is consistent with recent genetic and epidemiological findings. The shared familial tendency that transcends across diabetes phenotypes was highlighted by a Swedish population study that found children with type 2 diabetes in the parental lineage had nearly double the chance of T1DM (RR 1.88, p < 0.001).^13^ Furthermore, HLA and immune regulatory gene clusters, which together increase vulnerability to disease, provide a clear explanation for family aggregation.

Our study’s link between early cereal introduction and higher T1DM is consistent with important findings from studies on newborn diets. The TEDDY study later connected cereal introduction outside of the four to six month “window” with increased autoimmunity in genetically predisposed children, whereas the DAISY study indicated that introducing wheat or barley before four months of age increased islet autoimmunity risk.^14^ In line with our findings that early cereal introduction is more prevalent in T1DM cases, these investigations support the notion that introducing cereals too soon-or too late-may interfere with gut immune conditioning.

The introduction of the cereals before the sixth month^17^ and gluten before the age of four months^18^ had been linked to a higher incidence of type one diabetes. Children’s gluten consumption had an increased risk of T1DM, with an aHR of 1.46 (95% CI1.06-2.01, p =0.02) for every 10-g daily increase.^19^ It is anticipated that children will initially be introduced to wheat-containing cereals, including gluten, as they transition to complementary foods. The theory behind these findings was that a gluten-free diet might stop gliadin peptides from passing through the intestinal barrier by decreasing intestinal permeability, which would stop pancreatic autoimmunity from developing.^20^

### Limitations:

Inherent to case-control design, parental recall of possible exposures in our study particularly for older children was a major limitation. Because only one tertiary care hospital was used for the study, the findings might not apply to larger populations in other areas or medical settings. Maternal education, socioeconomic position, pregnancy nutrition, and history of other infections that may affect feeding habits and the risk of T1DM were not taken into consideration.

## CONCLUSION

This study highlights that early cereal introduction, female gender, mumps history, a positive family history of diabetes, and absence of exclusively breast feeding are all individually linked to a child’s higher risk of developing T1DM. In addition to targeted surveillance in at-risk paediatric populations for early prevention and intervention, these findings emphasise the need of encouraging exclusive breastfeeding and suitable weaning behaviours. In places where vaccine-preventable illnesses may still contribute to autoimmunity, mumps immunisation should be reinforced to ensure high coverage.

### Authors’ Contributions:

**MSM:** Conceived, designed, did statistical analysis & editing of manuscript.

**MZS:** Data collection and manuscript writing.

**PA:** Manuscript writing, and critical review of manuscript.

**AK:** Editing and review of manuscript.

All authors have read and approved the final version to be published and responsible for integrity of work.

***Conflict of interest:*** None.

***Source of funding:*** None.
